# Investigating Relationships between Reproduction, Immune Defenses, and Cortisol in Dall Sheep

**DOI:** 10.3389/fimmu.2018.00105

**Published:** 2018-01-31

**Authors:** Cynthia J. Downs, Brianne V. Boan, Thomas D. Lohuis, Kelley M. Stewart

**Affiliations:** ^1^Department of Biology, Hamilton College, Clinton, NY, United States; ^2^Department of Natural Resources and Environmental Sciences, University of Nevada, Reno, NV, United States; ^3^Alaska Department of Fish and Game, Anchorage, AK, United States

**Keywords:** allocation theory, carryover effect, constitutive immunity, glucocorticoids, immune defenses, *Ovis dalli dalli*, reproduction, trade-offs

## Abstract

Life-history theory is fundamental to understanding how animals allocate resources among survival, development, and reproduction, and among traits within these categories. Immediate trade-offs occur within a short span of time and, therefore, are more easily detected. Trade-offs, however, can also manifest across stages of the life cycle, a phenomenon known as carryover effects. We investigated trade-offs on both time scales in two populations of Dall sheep (*Ovis dalli dalli*) in Southcentral Alaska. Specifically, we (i) tested for glucocorticoid-mediated carryover effects from the breeding season on reproductive success and immune defenses during parturition and (ii) tested for trade-offs between immune defenses and reproduction within a season. We observed no relationship between cortisol during mating and pregnancy success; however, we found marginal support for a negative relationship between maternal cortisol and neonate birth weights. Low birth weights, resulting from high maternal cortisol, may result in low survival or low fecundity for the neonate later in life, which could result in overall population decline. We observed a negative relationship between pregnancy and bacterial killing ability, although we observed no relationship between pregnancy and haptoglobin. Study site affected bactericidal capacity and the inflammatory response, indicating the influence of external factors on immune responses, although we could not test hypotheses about the cause of those differences. This study helps advance our understanding of the plasticity and complexity of the immune system and provides insights into the how individual differences in physiology may mediate differences in fitness.

## Introduction

Life-history theory is fundamental to understanding how animals allocate resources among life-history traits including performance traits and physiological functions that contribute to survival (1, 2). Immediate trade-offs occur within a short span of time and, therefore, are more easily detected. Trade-offs, however, can also manifest across multiple stages of the life cycle, a phenomenon known as carryover effects (3). That is, events during one stage of the life cycle may influence allocation decisions of an individual during another stage of the life cycle. For example, capital breeders finance their reproduction from energy stores gained in months prior to the mating season (4, 5). Generally, individuals with larger fat stores upon initiation of reproduction have the greatest reproductive success (6) and those who initiate reproduction with insufficient energy stores pay a fitness cost of reduced offspring survival (3, 7, 8). Because fat reserves at the time of reproduction depend upon previously acquired resources, events that affect resource acquisition in one season carry over and subsequently affect the energy available for reproduction (9). Similar logic dictates that allocation of resources to physiological functions, such as immune defenses, may be influenced by carryover effects, and physiological mechanisms, such as integrative hormone signaling networks, may mediate these effects across seasons (10, 11).

Survival and reproduction are two key life-history traits, and selection should result in optimal investment in processes that contribute to both (1). Because immune defenses contribute to survival, energetic and nutritional costs of maintaining and mounting immune defenses are included within the optimality equation for life histories (12–14). The effects of collateral damage from immune responses, that is immunopathology, are also included within these optimality equations of life history (15, 16). As a consequence of the expense of immune defenses, individuals are often unable to maintain both a high level of immune function and successfully reproduce when limited resources force a trade-off in allocation between these two physiological processes (14, 15, 17, 18). Nonetheless, life-history strategy may dictate whether an individual reduces investment in reproduction in favor of immune defenses in a given year (16). Many species reduce immune defenses when committed to reproduction (17–22). However, manifestation of these trade-offs may depend on an individual’s energetic state and be regulated facultatively, and it may depend on overall life history strategy (16, 17). Large herbivores often exhibit tradeoffs between current and future reproduction, rather than between reproduction and survival (1, 23–25). Thus, individuals from slow-paced species should favor investment in traits that enhance survival, such as immune defenses, when resources are limited (6, 26, 27).

Availability of energetic and nutritional resources mediate trade-offs between immunological and reproductive performance mechanistically through integrative physiological networks and shared signaling molecules (15, 28). Specifically, key integrators in physiological pathways may act as the mechanism by which carryover effects mediate differences among individuals in investment in immune function and reproduction (11, 15, 29). Glucocorticoids are one proposed integrator of carryover effects (30). Glucocorticoids are released to support metabolically demanding activities and, as such, their circulating concentrations change during predictable seasonal and life-cycle events and they increase in response to stressful events (31–34). As part of the signaling pathway that mediates immune defenses and reproduction, acute increase of glucocorticoids facilitates reproduction and stimulate or redistribute immunological defenses (28, 33, 35, 36). However, sustained, elevated concentrations of glucocorticoids that are indicative of chronic stress can suppress immune defenses, prevent pregnancy, and lower juvenile survival by reducing birth weight of neonates (31, 35, 37–39). In brief, glucocorticoids can alter the cost-benefit equation of investment in immune defenses (28).

Changes in glucocorticoid levels during one stage of the life cycle may affect events during another stage (30). Specifically, high levels of glucocorticoids during mating may reduce reproductive success in large ungulates in two ways. First, integrated long-term levels of glucocorticoids may indicate elevated energy expenditure (40, 41), and females with high energy expenditures during the mating season may have fewer resources to invest in reproduction. Alternatively, high levels of glucocorticoids during the mating season may indicate chronic stress unrelated to energetic demands, and high, chronic stress levels can suppress reproduction regardless of energetic constraints (31, 33, 41).

During pelage growth, glucocorticoids from blood are deposited into hair of mammals and evidence suggest that hair glucocorticoids represents systemic-levels of circulating free glucocorticoids (42–44). Thus, glucocorticoid concentrations in pelage represent a non-invasive measure of integrated blood concentrations during pelage growth and provide researchers with the opportunity to study carryover effects by providing information about physiological state during a life event prior to sample collection (41, 45). Briefly, hair glucocorticoid concentrations are sensitive to major prolonged stressors and are correlated with changes in circulating glucocorticoids observed during pregnancy (43, 46, 47). However, single acute events of high glucocorticoids are not reflected in hair glucocorticoid concentrations (48). Thus, measurements of hair glucocorticoids provide a good indicator of energetically demanding or stressful events experienced during pelage growth (43). In most large mammals, pelage growth during the autumn occurs concurrently with the mating season, and thus provides an indicator of an individual’s state when allocation decisions about reproduction were made.

We studied two populations of Dall sheep (*Ovis dalli dalli*) in Southcentral Alaska to investigate (i) trade-offs between immune defenses and reproduction within a season and (ii) carryover effects mediated by glucocorticoids on reproduction in a long-lived, slow-paced species. Dall sheep are large ungulates that reside in highly seasonal environments throughout mountainous regions in Alaska and northwestern Canada. They produce a maximum of one offspring per year. We focused on an energy allocation framework because Dall sheep are capital breeders that live in a seasonal environment and, after mating in the fall, undergo gestation during the winter months when food is scarce. They undergo parturition beginning in May and lactate into the early autumn (49). Capital breeders like Dall sheep rely on stored energy reserves for the majority of their energy during the winter, their annual cycles are organized around gaining energy stores in the summer to fuel survival and reproduction in the winter, and their ecology makes energy trade-offs likely (50). We focused on constitutive immunity because these defenses are always present, they represent the first line of physiological defense against an invading pathogen, and they can mediate the outcome of some infections (51, 52). Specifically, we quantified bactericidal capacity and haptoglobin. Bactericidal capacity provides a broad assessment of host immune ability to eliminate bacterial pathogens (53, 54). Haptoglobin is a protein marker that is generally interpreted as a biomarker of an individual’s ability to upregulate inflammation (55). The inflammatory response is a cascade involving acute phase proteins that recruit cells and molecules to destroy pathogens (55–57).

Both energy constraints and elevated glucocorticoids can serve as mechanisms that underlie a potential carryover effect from autumn to spring that can reduce pregnancy and constitutive immune function. Thus, we expect that high glucocorticoid levels in the autumn will reduce the probability of parturition in the spring. The timing of our measurements means that our measure of reproduction integrates the probability of becoming pregnant, probability of successful parturition if pregnant, and probability of the neonate surviving until the time of capture. Specifically, we hypothesized (i) that individuals that are non-pregnant in the spring would have had higher glucocorticoid levels during the previous autumn than those that are pregnant in the spring and (ii) that within pregnant individuals, high glucocorticoid levels in the autumn would lead to reduced birth weights of neonates in the spring. Because we expect this slow-paced species to invest in survival (i.e., immune defenses) over reproduction, we did not expect pregnant individuals to reduce constitutive immunity relative to non-pregnant individuals.

## Materials and Methods

### Study Area

**Figure 1:**
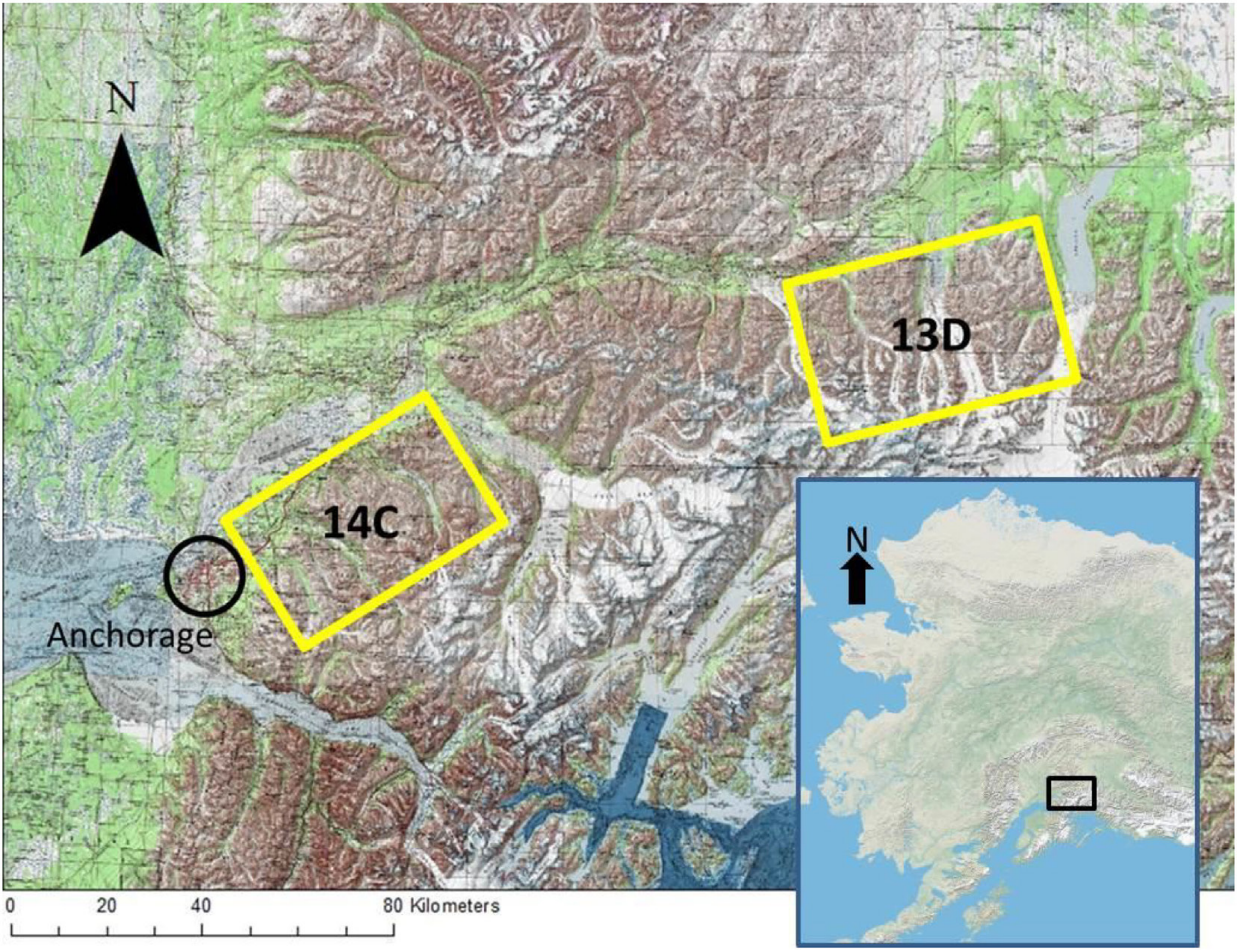
Map of study areas GMU 14C and GMU 13D in the Chugach Range, AK. Anchorage is within the black circle. The map inlay of Alaska shows approximate location of the study units within the state.

We studied two populations of Dall sheep in Southcentral Alaska in the Chugach range. One population was within Alaska Department of Fish and Game’s game management unit 14C (GMU 14C) and the other was within game management unit 13D (GMU 13D). The units are approximately 76.5 km apart and separated by a prominent glaciers (Figure 1). Dominant vegetation is similar in both study areas and changes with elevation; black spruce (*Picea mariana*) and alder (*Alnus* spp.) occur at lower elevations and short alpine forbs (e.g., mountain avens, *Dryas octopetala*) and grasses (e.g., alpine timothy, *Phleum alpinum*) occur at higher elevations. Most sheep in the study resided between 914 and 1,829 m in elevation.

GMU 14C lies within the Chugach State Park northeast of Anchorage and is comprised of five drainages and their surrounding ridges: Goat Creek, Eklutna, Peters Creek, Eagle River, and Ship Creek. GMU 14C is bordered by Anchorage and the town of Eagle River to the west, the Knik arm and river to the North, and the Turnagain arm to the South. The total study area is approximately 800 km^2^. From the weather station closest to GMU 14C, the 30-year (1981–2010) mean annual temperature was about 2.4°C, mean annual snowfall was 189.23 cm, and mean annual precipitation was 41.91 cm (58).

GMU 13D is northeast of GMU 14C and lies between the Matanuska Glacier and Tazlina Lake and is bordered to the north-northwest by the Alaska State Highway 1. GMU 13D is comprised of several mountain groups separated from each other by three prominent glaciers—Powell, Nelchina, and Tazlina—that limit movement within the study unit. The total study area is approximately 925 km^2^. From the weather stations closest to GMU13D, the 30-year (1981–2010) mean annual temperature was −2.17°C, mean annual snowfall was 167.13 cm, and mean annual precipitation was 41.33 cm (58).

### Field Methods

In 2012 and 2013, we captured sheep using standard helicopter and netgun techniques (59), and marked individuals with Telonics VHF radio collars. We attempted to recapture the same individuals each year, however, inaccessible terrain and weather conditions hindered capture efforts of some individuals. During capture, we collected blood samples (2012 and 2013) to determine pregnancy status and to quantify immune defenses, and hair samples (2013 only) to quantify cortisol levels. We determined pregnancy by testing blood serum for the presence of pregnancy specific protein B (BioTracking LLC, Moscow, ID, USA) (25, 60, 61). We determined age using horn growth rings (62). A body condition score was assigned based on the amount of bony structural protrusions felt in the rump, spine, neck, and shoulders (63). Scores ranged from 0 to 5 with 0 representing no subcutaneous fat and five representing individuals with substantial fat reserves (63). All radio-tagged animals were monitored once to twice per week from March to early May when parturition occurred. We attempted to monitor females every day during the parturition season (approximately May 7 to June 10) to detect and capture neonates. Neonates were captured on the ground on foot, collared with Telonics VHF radio collars, weighed (to the nearest 0.1 kg), and sex was determined. Age in days was estimated based on umbilical cord presence and condition, pelage coloration, and mobility. As neonates age, the umbilical cord dries and usually has fallen off by 3 days of age (T. Lohuis, personal observation). At the time of birth, neonate pelage has a gray appearance and lightens with age (T. Lohuis, personal observation). In addition, Dall sheep neonates are precocial and quickly gain stability and mobility with increasing age (64, 65), and we were often limited to capturing neonates between 0 and 5 days old because of this. We did not recapture adult females at the same time that we captured neonates.

### Ethical Statement

All aspects of this research were approved by the Institutional Animal Care and Use Committee at the University of Nevada Reno (Protocol #2012-00542) and Alaska Department of Fish and Game (Protocols #2009-13 and #2012-024). All methods were in keeping with protocols adopted by the American Society of Mammalogists for field research involving mammals (66). Use of *Escherichia coli* was approved by the Institutional Biosafety Committee at University of Nevada Reno (B2013-10) and methods were in keeping with recommendations in CDC/NIH Guidelines.

### Immune Assays

The bacteria killing ability assay was used to quantify bactericidal capacity of circulating, constitutive components of immune defenses in plasma—complement, acute phase proteins, and natural antibodies (54, 67). We performed assays on serum samples, following Zysling et al. (68) and calibrated for Dall sheep. We used *E. coli* (Epower Microorganisms #0483E7, ATCC8739, MicroBioLogics, St. Cloud, MN, USA) as our ecologically relevant pathogen. Briefly, we mixed 100 µl of a sample with 100 µl of Luria Bertani (LB) broth to make a 1:2 dilution of serum solution and then added 20 µl of our working *E. coli* solution (~5,000 bacteria ml^−1^). We made two positive controls by mixing 200 µl LB broth with 20 µl of working *E. coli* solution. Samples and controls were vortexed and incubated at 37°C for 30 min. We vortexed samples and controls and plated 50 µl aliquots onto LB agar in petri dishes in triplicate to help ensure reproducibility. One positive control was plated at the beginning and end of each batch. Plates were incubated overnight at 37°C, after which bacteria colonies were counted. We used all six positive control plates (three replicates of two controls) to determine the mean number of control colonies for a particular batch, and samples were compared with the positive controls from their batch. Bactericidal capacity was calculated as percent bacteria killed relative to the positive control. To better ensure reproducibility, we calculated mean intra-control coefficient of variance (CV); it was 8.8%. It was inappropriate to look at CV of number of colony forming units on sample plates because small changes in the number of colonies among plates when there are few colonies results in very high CVs.

We measured haptoglobin concentrations in serum to assess potential for an inflammatory response. Functionally, haptoglobin is an acute phase protein that binds to heme preventing it from serving as a nutrient for pathogens and initiating deleterious oxidation reactions resulting in a rapid inflammatory response (55, 56, 69). Haptoglobin is normally present at low constitutive levels but increases when a pathogen is encountered (70). Because constitutive haptoglobin concentrations are predictive of haptoglobin concentrations after an endotoxin challenge (55), they are indicative of ability to mount an inflammatory response. We assessed haptoglobin presence in raw serum samples (i.e., not diluted) using the Phase Range Haptoglobin Assay Cat. N. TP-801 (Second Generation; Tridelta Development Ltd., Maynooth, Ireland) (55, 71). We followed the manufactures instructions. We ran 16 samples twice to check for reproducibility, the mean CV for these was 13.4%. Mean intra-sample CV was 2%.

### Cortisol Assays

We quantified cortisol levels from hair samples collected from 44 individuals in 2013. Cortisol is the dominant glucocorticoid in bighorn sheep (*Ovis canadensis*), another wild sheep species (72). To prepare hair for cortisol extraction, the hair was washed twice in isopropanol, dried, and ground to a powder using a ball mill (SPEX SamplePrep 8000M Mixer/Mill, Metuchen, NJ, USA). Ground hair samples were ~0.05 g and weighed to the nearest 0.0001 g. We then added 1 ml of methanol to each sample, rocked samples for 24 h, and centrifuged them at 2,000 rpm for 1 min. We pipetted 0.6 ml of the supernatant into a vial, evaporated the sample on a heat block at 37°C under nitrogen. The residual cortisol from the sample was reconstituted with 1,040 µl of a 95% assay diluent from the kit and 5% methanol mixture. We quantified cortisol levels using a commercially available kit (Salametrics Salivary Cortisol EIA kit, product #1-3002) (46), following modifications for hair samples suggested by Davenport et al. (46) and Koren et al. (42). Before quantifying samples, we verified that the kit worked for hair samples from Dall sheep by checking for parallelism between an unknown dilution curve and the standard curve (73). We used a high and low standard provided with the kit as an inter-assay control and we re-quantified all the samples on a plate if these controls were not within the expected range. We re-quantified samples if the coefficient of variation between replicates was greater than 15%.

### Statistical Methods

All statistics were performed in SAS (v. 9.3, SAS Inst 2010). We assessed variation in pregnancy rates between years and study sites using the *Z*-test for proportions that allowed sampling with replacement (74). The *Z*-test was appropriate because we attempted to capture the same individuals each year, and no new animals were added to the study after the first year of capture. We used Pearson correlations (Proc CORR) to determine the repeatability of our immune measure across years and to determine the correlation between our immune measures within year. We used untransformed data for all correlations.

We determined the effect of pregnancy status on bactericidal capacity and haptoglobin concentrations using generalized linear models (Proc GLM), with a separate model for each year. We analyzed each year separately because not all individuals were captured in both years. We transformed bactericidal capacity from percent to angular arcsine square root to improve normality of residuals (74). We included study site and body condition score as main effects for both bactericidal capacity and haptoglobin concentration. To account for possible senescence, we included age as a predictor variable of bactericidal capacity and haptoglobin concentrations using linear regression weighted by sample size (74). We did not find a relationship and, therefore, did not include age in further analyses of constitutive immunity.

To determine the effect of cortisol during the autumn on pregnancy status (binomial response: pregnant or not pregnant) the following spring, we used logistic regression (Proc Logistic) and included study site and spring body condition score as dependent variables alongside cortisol. During the preliminary analysis, we also included age as a predictor variable of pregnancy but did not find a relationship and, therefore, did not include age in the final analysis.

To calculate neonate birth weight, we used linear regression to estimate weight gained per day and back calculated birth weight based on age and weight of the neonate at capture. We then determined the effect of cortisol during the autumn on neonate birth weight during the spring with analysis of covariance (Proc GLM). We included maternal age, maternal body condition during the spring, study site, and cortisol concentration as explanatory variables. We also determined the effects of a maternal haptoglobin or bactericidal capacity during pregnancy on birth weights of neonates using separate linear regressions (Proc REG). Significance was determined at α = 0.05; however, we consider patterns intriguing at α = 0.1 because of our small sample sizes.

## Results

**Figure 2:**
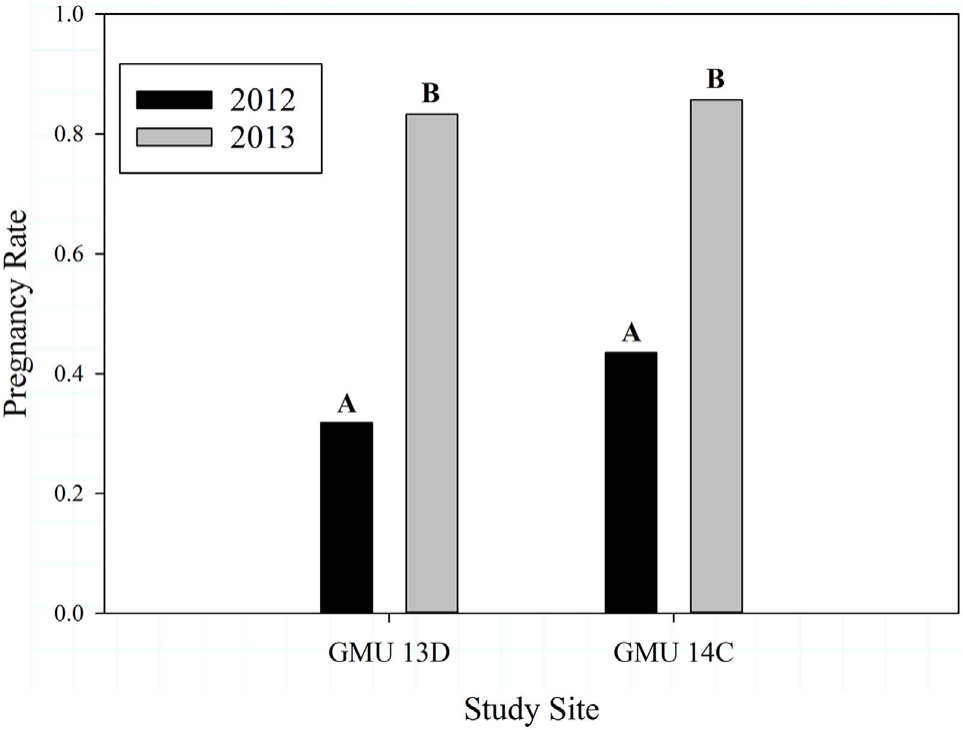
Dall sheep pregnancy rates for years 2012 and 2013 (*Z* = 4.31, *P* < 0.0001) and study sites (2012 Z = 0.503, *P* = 0.615; 2013 *Z* = −0.092, *P* = 0.927) in the Chugach Range, AK. Letters over bars indicate results of comparisons following a significant *Z*-test, where different letters are statistically different.

The number of adult female sheep and lambs we captured for each GMU during 2012 and 2013 are presented in Table 1. Adult female ages ranged from 2 to 11 years and body condition scores ranged from 1.75 to 2.75. Overall pregnancy rates were higher in 2013 (0.833) than in 2012 (0.378) (*Z* = 4.31, *P* < 0.0001), and this pattern held for both GMUs (Table 1; Figure 2). Pregnancy rates did not differ between study sites in either year (2012: *Z* = 0.503, *P* = 0.615; 2013: *Z* = −0.092, *P* = 0.927, Figure 2).

**Table 1:** Description of number of adult female and neonate Dall sheep captured in each of the study areas (GMU) for each year in the Chugach Range, AK 2012–2013.

Year	GMU	Adult females captured	Pregnancy rate	Neonates captured
2012	13D	31	0.32	14
	14C	34	0.44	26

2013	13D	26	0.83	26
	14C	22	0.86	11

### Immune Defenses

We had bactericidal capacity data from 91 samples and haptoglobin data from 87 samples; our samples were spread fairly evenly among sites and years (Table 2). Haptoglobin had an 18% repeatability when data were combined across sites, but was not repeatable when only 13D or 14C were examined independently. Bactericidal capacity had a 53% repeatability in GMU 14C, but was not repeatable across years in 13D or when all data were combined (Table 3). Haptoglobin levels were not correlated with bactericidal capacity within a year for either site or all the data combined (Table 3).

**Table 2:** Samples sizes for immune assays and the cortisol assay by year and site in the Chugach Range, AK 2012–2013.

Site	Year	Bacteria killing assay	Haptoglobin	Cortisol
All data		91	87	44
14C	2012	22	21	N/A^a^
	2013	24	25	22
13D	2012	23	20	N/A
	2013	22	21	22

*^a^We did not quantify cortisol concentrations in 2012*.

**Table 3:** Repeatability of immune assays between years and correlations between immune traits within years for all data combined and data from each study areas (GMU).

Sites	Variable 1	Variable 2	*r*	*P*-value	*n*
Both GMUs	BKA 2012	BKA 2013	0.258	0.176	29
	**Hapt 2012**	**Hapt 2013**	**−0.180**	**0.036**	**28**
	BKA 2012	Hapt 2012	−0.015	0.938	28
	BKA 2013	Hapt 2013	−0.062	0.748	29
13D only	BKA 2012	BKA 2013	0.126	0.967	13
	Hapt 2012	Hapt 2013	−0.308	0.263	15
	BKA 2012	Hapt 2012	−0.058	0.845	14
	BKA 2013	Hapt 2013	−0.119	0.687	14
14C only	**BKA 2012**	**BKA 2013**	**0.532**	**0.034**	**16**
	Hapt 2012	Hapt 2013	0.485	0.093	13
	BKA 2012	Hapt 2012	0.213	0.465	14
	BKA 2013	Hapt 2013	0.100	0.724	15

*BKA, bactericidal capacity assay; Hapt, haptoglobin*.

*Bold results indicate significant correlations*.

**Figure 3:**
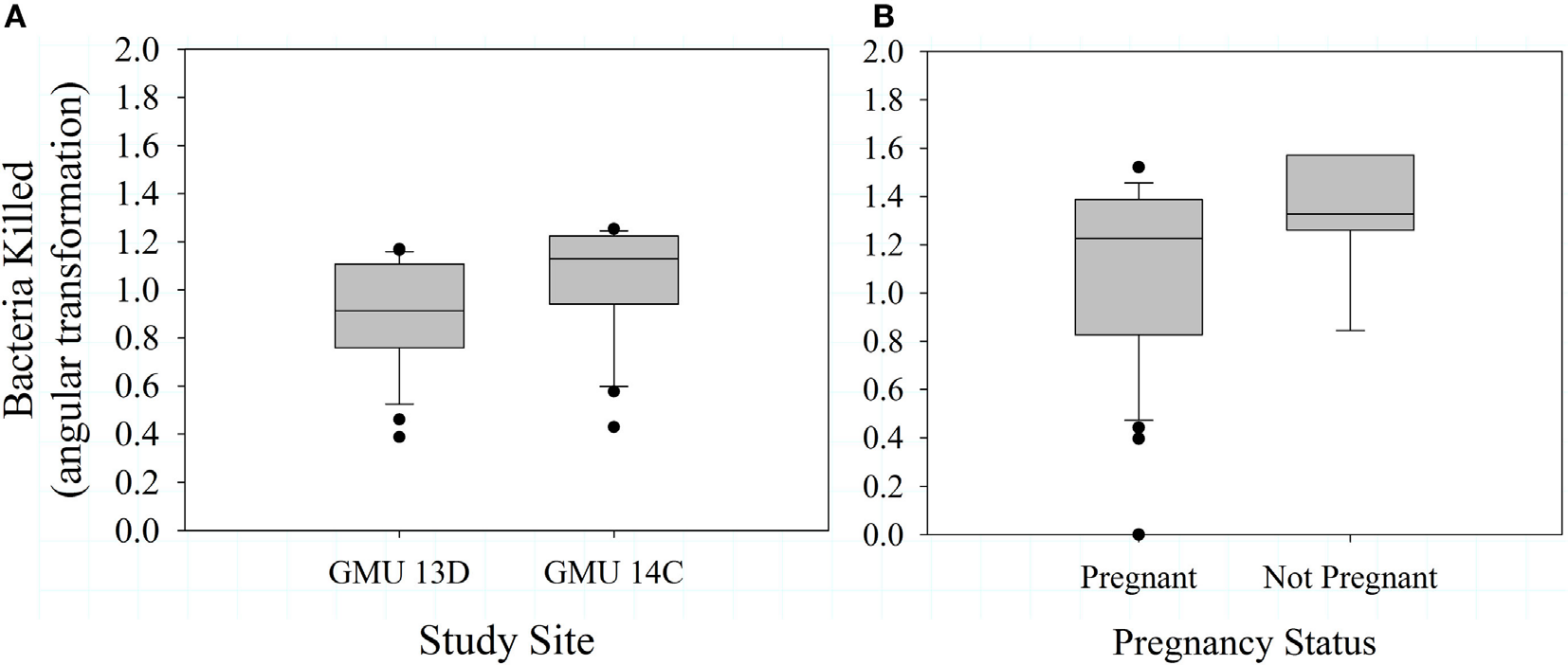
Mean (±SE) bactericidal capacity for female Dall sheep by study sites in the Chugach Range, AK **(A)** and pregnancy status **(B)**. Samples were collected in 2013.

Mean bactericidal capacity was 74.0 ± 27.4% during 2012 and 76.2 ± 27.7% during 2013. In 2012, bactericidal capacity was not associated with pregnancy status (*F*_1,45_ = 0.52, *P* = 0.476), body condition (*F*_3,40_ = 0.200, *P* = 0.896), or study site (*F*_1,45_ = 2.41, *P* = 0.128). Similarly, we observed no relationship between body condition and bactericidal capacity in 2013 (*F*_2,41_ = 0.690, *P* = 0.507). In 2013, however, bactericidal capacity was significantly greater in GMU 14 C than GMU 13D (*F*_1,45_ = 5.050, *P* = 0.030; Figure 3A), and there was an intriguing pattern of a negative effect of pregnancy status on bacterial killing ability (*F*_1,45_ = 3.19, *P* = 0.081; Figure 3B; overall model *F*_2,45_ = 3.99, *P* = 0.026). Bactericidal capacity was not significantly associated with neonate birth weight (*F*_1,13_ = 1.89, *P* = 0.193, *n* = 15).

**Figure 4:**
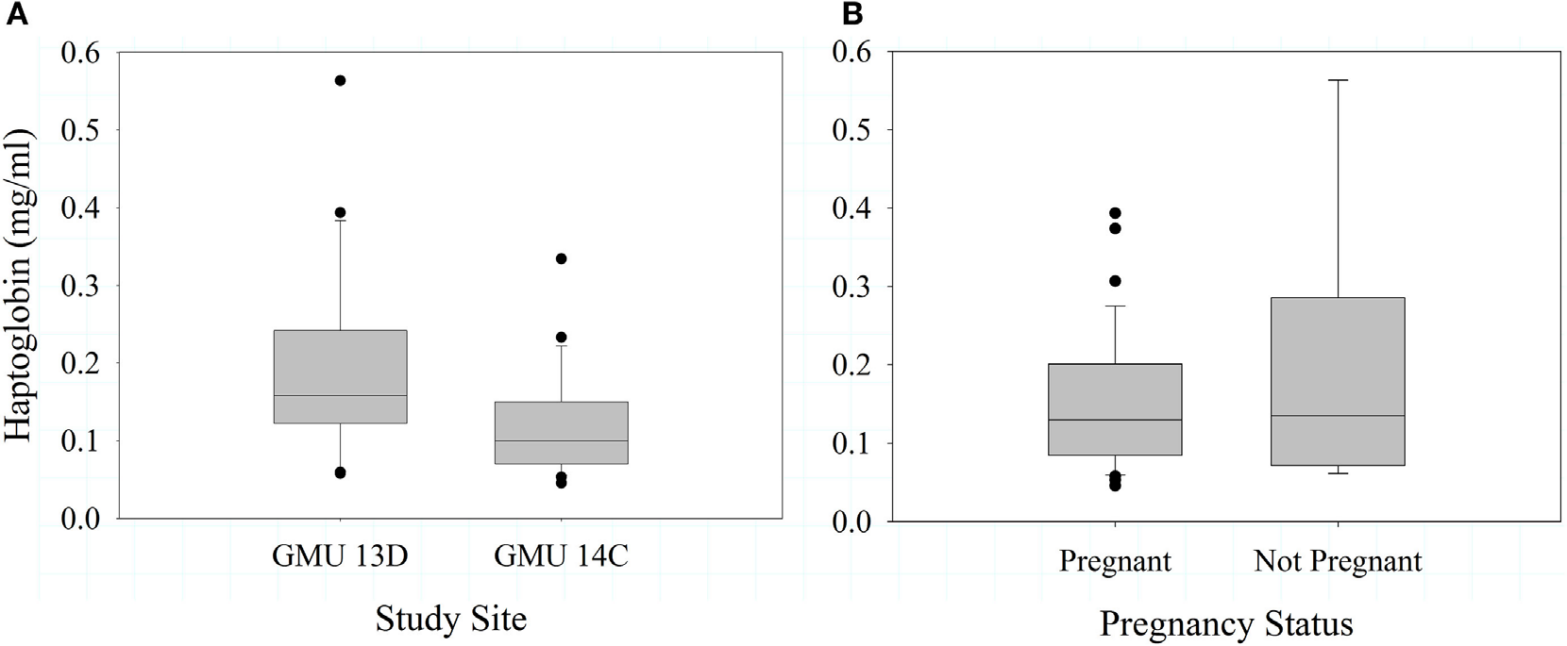
Mean (±SE) haptoglobin concentrations in female Dall sheep by study sites in the Chugach Range, AK **(A)** and pregnancy status **(B)**. Sample were collected 2013.

In 2012, haptoglobin did not differ between sites (*F*_1,41_ = 0.79, *P* = 0.381), and was not associated with pregnancy status (*F*_1,45_ = 1.16, *P* = 0.287) or body condition (*F*_2,41_ = 0.180, *P* = 0.832). In 2013, haptoglobin concentration was significantly greater in GMU 13D than 14C (*F*_1,46_ = 4.64, *P* = 0.037, Figure 4A); this is the opposite of the pattern observed for bactericidal capacity between sites. In 2013, haptoglobin was not significantly predicted by pregnancy status (*F*_1,41_ = 0.45, *P* = 0.506, Figure 4B) or body condition (*F*_3,37_ = 0.630, *P* = 0.598). Haptoglobin levels were not significantly associated with neonate birth (*F*_1,13_ = 0.89, *P* = 0.362, *n* = 15).

### Cortisol

**Figure 5:**
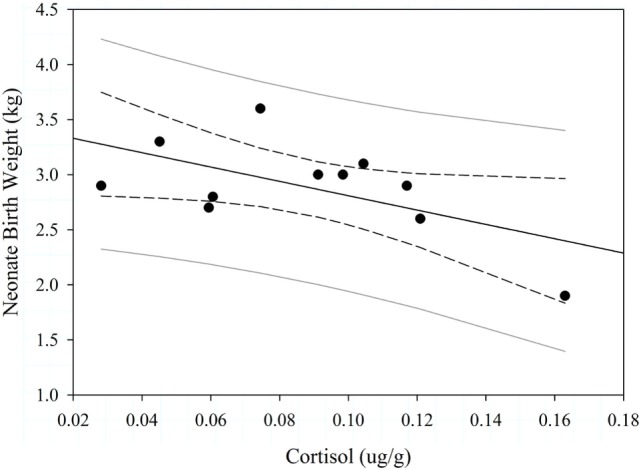
Effect of maternal cortisol levels on Dall sheep neonate birth weight (*n* = 11) in the Chugach Range, AK, 2013. The dashed lines are the 95% confidence limits and gray solid lines are the 95% prediction limits.

We quantified concentrations of cortisol from 44 samples; those samples were evenly distributed between the two study sites (Table 2). In 2013, logistic regression was 65.8% concordant and indicated that pregnancy status during the spring was not associated with cortisol during the previous autumn (Wald Chi-Square = 1.275, *P* = 0.259), body condition during the spring (Wald Chi-Square = 1.331, *P* = 0.249), or study site (Wald Chi-Square = 0.008, *P* = 0.9298). There was a marginal negative trend between maternal cortisol during the autumn mating season and neonate birth weight the following spring (*F*_1,11_ = 4.79, *P* = 0.057, Figure 5). Adult age (*F*_1,11_ = 1.000, *P* = 0.547), spring body condition (*F*_1,11_ = 2.70, *P* = 0.146), and study site (*F*_1,11_ = 1.14, *P* = 0.317) did not explain birth weight of neonates during spring.

## Discussion

We tested for glucocorticoid-mediated carryover effects on reproductive success. We also tested for trade-offs between immune defenses and reproduction within a season. We observed no relationship between maternal cortisol and pregnancy success; however, we found marginal support for a negative relationship between maternal cortisol and neonate birth weights, providing weak evidence of a carryover effect. We observed a weak negative relationship between pregnancy and bacterial killing ability during 1 year of our study providing evidence of a trade-off, although we observed no relationship between pregnancy and haptoglobin. We also found that constitutive immunity differed between sites and years highlighting the plasticity of immune defenses.

### Carryover Effects Mediated by Cortisol

We found no evidence of cortisol mediating the ability to become pregnant during the mating season in autumn 2012. Because pregnancy rates were low in the spring of 2012, few sheep underwent the energetically expensive processes of gestation, parturition, and lactation prior to the 2012 autumn mating season. Females in autumn 2012 were likely in better condition going into the mating season because of so few individuals produced offspring the previous year (6, 23). Because females in good nutritional condition are more likely to conceive and maintain pregnancy (6, 23), the low pregnancy rate in 2012 may have led to the high pregnancy rate of spring 2013. If our interpretation is correct, these data would provide evidence of a carryover effect between years, and they corroborate a pattern commonly found in capital breeders with large body sizes (23–25).

Cortisol levels observed during autumn may not have been high enough to affect pregnancy, but we found weak evidence that they were high enough to affect fetal development. Even though we had a small sample of body weights of neonates, we observed that cortisol levels of mothers during autumn were weakly and inversely correlated with birth weights of neonates born the following spring. The effect of maternal cortisol during mating on offspring mass potentially has longer-term implications of reducing success of offspring because low birth weight has been shown to reduce survival of young and lower fecundity during adulthood (75, 76). Furthermore, in highly seasonal environments, like Alaska, young with low birth weight have rarely been observed to catch up in body mass with the larger individuals in their cohorts (77, 78), and larger adults tend to have higher fitness (79). Therefore, the disadvantage of being born small may carry throughout the individual’s lifetime.

### Pregnancy and Immune Defenses: Evidence of a Carryover Effect?

Parturition in Dall sheep occurs in June. We captured adult female sheep in mid-March, and during that stage of the annual cycles, females are not provisioning offspring (i.e., lactating) from the previous year and because lactation is energetically expensive and most are relying on stored fat for energy because environmental food sources are not readily available (80). Although pregnancy status was not significantly associated with either measure of constitutive immunity in 2012, we observed an interesting trend in 2013, whereby sheep that were pregnant had lower bactericidal capacity than those that were not pregnant (*P* < 0.1). Although our sample sizes were small, the trend suggesting a relationship between pregnancy and bactericidal capacity is worth examining further in future studies.

If generalizable, this trend suggests that Dall sheep approaching parturition invest fewer resources in immune response in favor of reproduction, as has been seen in numerous fast-paced species (17–21). Published results about trade-offs between immune defenses and pregnancy in slow-paced ungulates are ambiguous. Pregnant N’Dama cows maintained under traditional husbandry practices in Gambia had higher rates of Trypanosomiasis than non-pregnant cows (81), indicating a difference in underlying physiology or behavior. Similarly, free-ranging Soay sheep (*Ovis aries*) with higher concentrations of antinuclear antibodies, an indicator of immune defenses, had lower siring probability and lower female breeding probability (82), although this study is not a direct comparison to ours because we instigated how pregnancy affected constitutive immunity rather than investigating how constitutive immunity affected probability of pregnancy. In contrast, pregnancy status did not affect hemolytic-complement activities or bactericidal activity in free-ranging North American elk (*Cervus elaphus*) (26), nor did it affect bactericidal capacity in free-ranging African buffalo (*Syncerus caffer*). These contradictory results suggest that an immune trade-off with reproduction may depend on the immune defense measured, timing of the measurement, and other external variables that regulate traits such as physiological condition and hormone concentrations. Mechanistically, and as previously discussed, the negative trend between pregnancy and immune defenses in 2013 could arise from a carryover effect that is likely mediated by an individual’s energy budget.

As pregnancy progresses, some studies show that constitutive immune defense decline (83, 84). Other studies have shown that immunity shifts from humoral to cell-mediated responses as pregnancy progresses (85–87), although those studies focused on adaptive rather than innate immune responses. Our results are consistent with both of those patterns, because we found that humoral measures of constitutive immunity were lower in sheep late in their pregnancy. These shifts in immune strategy might be facilitated by hormones that mediate pregnancy. The physiology mediating pregnancy alone, however, is unlikely to explain our results because we did not find a significant difference in immune defenses between pregnant and non-pregnant sheep in both years. That is, our incontinent results over years may indicate a facultative strategy caused by a reconfiguration of the immune system to increase probability of a successful reproductive event while taking into account other cues such as body condition (17, 28, 87).

### Population and Individual-Level Patterns in Immune Defenses

Immune defenses are highly plastic and change between years, seasons, and even weeks (88–90). Immune assays in our study exhibited repeatability across years for only 2 of the 8 analyses we performed, and those repeatabilities suggested an upper limit of additive genetics of only 54 and 18% (Table 3). Similarly, a study of captive red knots (*Calidris canutus*) showed that microbiocidal activity for three microbes, white blood cell differentials, hemolytic and hemogglutination activity changed across the annual cycle (88). In addition, T-cell-mediated immunocompetence of free-ranging, nesting tress swallows was influenced by weather within a single nestling season (89).

Constitutive bactericidal capacity and constitutive haptoglobin concentrations of plasma differed between sites during 2013, and interestingly, bactericidal capacity and haptoglobin concentrations showed opposite trends between the two sites. In 2013, sheep from GMU 13D had higher haptoglobin concentrations and lower bactericidal capacity relative to sheep from GMU 14C. These results add to a growing number of studies that have found population-level differences in constitutive immunity. At the same spatial scale of our study, other studies have linked differences in constitutive immunity to group-level differences in mean physiological state (26), parasite prevalence (91), population characteristics such as density (26, 92), habitat quality (92, 93), and weather (89). We cannot distinguish among these alternative possibilities with our data.

Interestingly, neither immune response was related to body condition as measured by body condition scores. These results have two possible explanations. First, body condition scores alone are a coarse measure of body condition and likely are subject to observer bias (94, 95). Therefore, body condition score may not be a precise enough measure to correlate with physiological traits, like immune defenses. Second, we observed little variation in body condition scores. The range of fat scores was 1.75–2.75 on a 6-point scale, and thus the sheep were generally in medium to low nutritional condition, and none were in high condition (63). The range of condition scores is likely related to the season in which we captured individuals. In strongly seasonal environments, as in our study, all individuals lose weight and decline in body condition even when fed *ad libitum* because they use stored fat for energy (96). At the time of capture, sheep were likely in the lowest condition of the annual cycle because we captured at the end of winter (80).

## Conclusion

Our correlative results suggest extrinsic factors, including study site, play a larger role in mediating immunocompetence than the intrinsic factors studied (reproduction and body condition). This finding corroborates previous work that found local environmental conditions can overwhelm the genetic signature of immune defenses (89). We found weak evidence that high maternal cortisol levels are correlated with low neonate birth weights. Thus, our results have implications for understanding how individual-level differences in chronic glucocorticoids concentrations and individual-differences in immune defenses may affect population dynamics by affecting fitness components (97).

Although interpretation of our results is limited by the complexity of a natural system and the difficulties of studying a natural system in rough terrain and harsh weather conditions, we argue, as others do (98, 99), that studying immunological defenses in free-ranging animals is important for understanding dysfunction of the immune system. Immune defenses and pathologies are the product of genotype by environment interactions and these interactions are hard to replicate in laboratory conditions (100–103). For example, a high level of pathogen exposure causes wild mice (*Mus musculus domesticus*) to have immune systems that are in a highly activated or primed state relative to those of laboratory mice (104). Furthermore, the implications for population dynamics are hard to extrapolate from laboratory studies. In general, lessons about immunological responses in wild, free-ranging animals will help advance our understanding of the plasticity and complexity of the immune system and enhance our understanding of disease and its effects on population dynamics.

## Ethics Statement

All aspects of this research were approved by the Institutional Animal Care and Use Committee at the University of Nevada Reno (Protocol #2012-00542) and Alaska Department of Fish and Game (Protocols #2009-13 and #2012-024). All methods were in keeping with protocols adopted by the American Society of Mammalogists for field research involving mammals (Sikes, Gannon & Amer Soc 2011). Use of *Escherichia coli* was approved by the Institutional Biosafety Committee at University of Nevada Reno (B2013-10) and methods were in keeping with recommendations in CDC/NIH Guidelines.

## Author Contributions

TL and KS designed the field component of the study and obtained funding for the project. CD, KS, and BB designed the laboratory portion of the study. BB and TL conducted field work and collected samples for physiological assays. CD and BB calibrated assays for Dall sheep, and BB performed all laboratory assays, CD, BB, and KS analyzed those data and wrote the manuscript. All authors contributed substantially to editing the manuscript and approved the final version.

## Conflict of Interest Statement

The authors declare that the research was conducted in the absence of any commercial or financial relationships that could be construed as a potential conflict of interest.
